# Transcriptional dysregulation of 5-HT1A autoreceptors in mental illness

**DOI:** 10.1186/1756-6606-4-21

**Published:** 2011-05-27

**Authors:** Paul R Albert, Brice Le François, Anne M Millar

**Affiliations:** 1 Ottawa Hospital Research Institute (Neuroscience), University of Ottawa, 451 Smyth Road, Ottawa, Ontario, K1H 8M5, Canada

## Abstract

The serotonin-1A (5-HT1A) receptor is among the most abundant and widely distributed 5-HT receptors in the brain, but is also expressed on serotonin neurons as an autoreceptor where it plays a critical role in regulating the activity of the entire serotonin system. Over-expression of the 5-HT1A autoreceptor has been implicated in reducing serotonergic neurotransmission, and is associated with major depression and suicide. Extensive characterization of the transcriptional regulation of the 5-HT1A gene (HTR1A) using cell culture systems has revealed a GC-rich "housekeeping" promoter that non-selectively drives its expression; this is flanked by a series of upstream repressor elements for REST, Freud-1/CC2D1A and Freud-2/CC2D1B factors that not only restrict its expression to neurons, but may also regulate the level of expression of 5-HT1A receptors in various subsets of neurons, including serotonergic neurons. A separate set of allele-specific factors, including Deaf1, Hes1 and Hes5 repress at the HTR1A C(-1019)G (rs6295) polymorphism in serotonergic neurons in culture, as well as *in vivo*. Pet1, an obligatory enhancer for serotonergic differentiation, has been identified as a potent activator of 5-HT1A autoreceptor expression. Taken together, these results highlight an integrated regulation of 5-HT1A autoreceptors that differs in several aspects from regulation of post-synaptic 5-HT1A receptors, and could be selectively targeted to enhance serotonergic neurotransmission.

## Serotonin in Major Depression

Major depression is a common and severe mental illness with a lifetime prevalence of 15% (1 in 6) compared with 1% for schizophrenia, and is twice as frequent in women as in men [1,2]. In developed countries, MDD currently accounts for the second highest lifetime burden of disease, and is forecast to be highest by 2030 [3-7]. With current antidepressant treatments, although up to 60% of patients respond, only 30% go on to remission [8-13], and 15% attempt suicide [14,15]. Although other neurotransmitters (e.g., noradrenaline, dopamine, glutamate, neurotrophins) are indirectly involved in depression [16-21], multiple lines of evidence implicate reduced 5-HT neurotransmission as a primary defect in depression [22-30]. For example, acute tryptophan depletion triggers relapse in recovered depressed patients, and elicits a depressed mood in normal subjects, while most antidepressant treatments, including serotonin-selective reuptake inhibitor (SSRIs), increase 5-HT neurotransmission either directly or indirectly [20,31-34]. Alterations in 5-HT1A receptor levels are commonly observed in depressed individuals. In particular, post-synaptic 5-HT1A receptors are reduced in several cortical regions in depression [35-39] and anxiety [40-45], while 5-HT1A autoreceptors are increased in depression [46-48]. Elevated 5-HT1A autoreceptor expression would tend to reduce the activity of 5-HT neurons, while reduced post-synaptic 5-HT1A receptors would result in a blunted behavioral response to 5-HT. These studies implicate the 5-HT1A receptor as an important determinant of predisposition to mental illness. However, the mechanisms underlying these differential changes in 5-HT1A receptor expression remain unclear. This review examines the evidence that alterations in transcriptional regulation of the 5-HT1A receptor could underlie its dys-regulation in mental illness.

## 5-HT1A receptors and the 5-HT system

### 5-HT1A autoreceptor function

**Figure 1:**
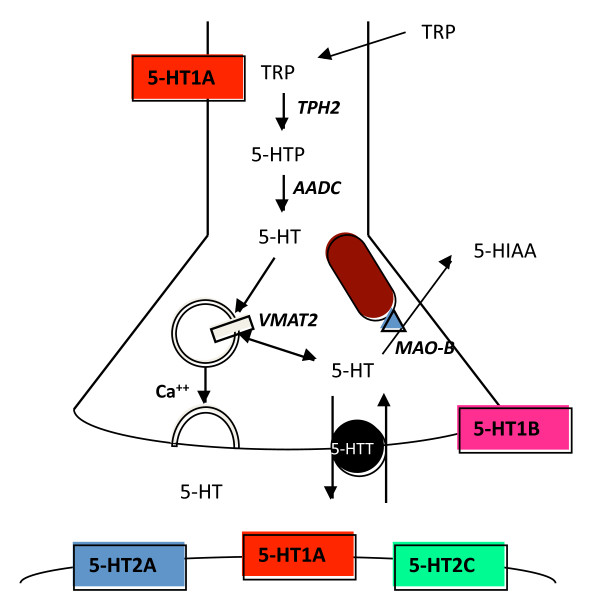
**Components of 5-HT neurotransmission**. Shown are the major components involved in the synthesis, vesicular packaging, reuptake, and degradation of serotonin in the brain, and the major receptors that mediate pre- and post-synaptic regulation of 5-HT neurotransmission. TRP, tryptophan; 5-HTP, 5-hydroxy-TRP; 5-HIAA, 5-hydroxy indole acetic acid; TPH2, tryptophan hydroxylase-2; AADC, aromatic amino-acid decarboxylase; VMAT2, vesicular monoamine transporter-2; MAO-B, monoamine oxidase B; 5-HTT, 5-HT transporter.

**Figure 2:**
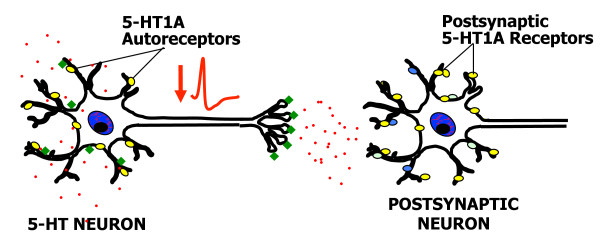
**5-HT1A autoreceptor-mediated negative feedback on 5-HT neurons**. A model of a serotonergic neuron (5-HT neuron) projecting to a target neuron (postsynaptic neuron) is shown, with 5-HT1A receptors depicted as yellow ovals, and other 5-HT receptors as other colored ovals. Acutely, SSRI's rapidly enter the brain and block 5-HT transporters (green diamonds), located at the raphe serotonin nerve terminal, but also at the cell body and dendrites in the raphe nuclei. This block of reuptake leads to accumulation of extracellular 5-HT (red dots) at both sites once released by depolarization. Activation of 5-HT1A autoreceptors located at the cell body and dendrites leads to inhibition of neuronal firing rate (red action potential), thus compensating for increase in 5-HT induced by SSRI treatment, resulting in little change in 5-HT neurotransmission initially.

The brain 5-HT system originates from neurons of the raphe nuclei that express tryptophan hydroxylase 2 (TPH2), the rate-limiting enzyme for 5-HT synthesis in the central nervous system [49-51] (Figure 1). These neurons project widely throughout the brain to regulate many functions, including sleep, mood, and stress reactivity [52-58] and are implicated in mental illnesses, including major depression and anxiety [26,27,55,57,59]. Among the 14 5-HT receptor genes [60], the 5-HT1A receptor is of particular interest since it is abundant in corticolimbic regions that are implicated in mood and emotion, such as the hippocampal and cortical pyramidal neurons and interneurons of the prefrontal cortex, medial septum, amygdala, hypothalamus, and other regions [60-64]. Presynaptically, the 5-HT1A receptor is the major somatodendritic autoreceptor on 5-HT neurons [65-67] where it acts as a "brake" to inhibit the activity of the entire 5-HT system and is thought to delay antidepressant response [68-74] (Figure 2). Hence mechanisms that regulate 5-HT1A autoreceptor levels are likely to set the tone of the entire 5-HT system and thus influence susceptibility to mood disorders such as depression, anxiety, and related disorders.

### Signaling of the 5-HT1A autoreceptor

**Figure 3:**
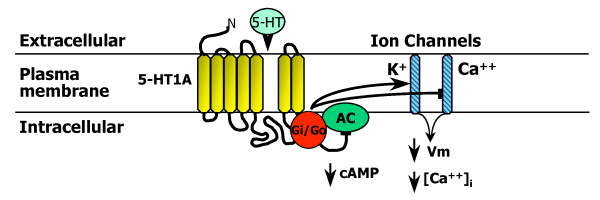
**Neuronal signaling of the 5-HT1A autoreceptor**. The major signaling pathways of the 5-HT1A receptor in neurons are shown. The 5-HT1A receptor is shown figuratively as a heptahelical G-protein coupled receptor that couples via inhibitory G proteins (Gi/Go) to inhibit adenylyl cyclase (AC) and reduce cAMP levels; to open G-protein inward rectifying potassium channels (K^+^) to reduce membrane potential (Vm); and to inhibit voltage-gated calcium channels (Ca^++^) and reduce intracellular free calcium concentration ([Ca^++^]_i_).

The 5-HT1A receptor was the first 5-HT receptor cloned and encodes a protein with seven hydrophobic transmembrane domains, typical of G-protein coupled receptors [61,75] (Figure 3). The 5-HT1A receptor couples to Gi/Go proteins, and in most cells and inhibits adenylyl cyclase activation, reducing cAMP levels [76,77]. Several studies have investigated the signaling of the 5-HT1A autoreceptor in raphe neurons [78]. This is also the case in the raphe nucleus, where the 5-HT1A autoreceptor preferentially couples to Gαi3 [79,80], and negatively regulates serotonergic neuronal activity, in part by inhibiting adenylyl cyclase [80-83]. Interestingly, signaling to this pathway depends on the 5-HT1A agonist used [82,84,85]. In addition, the 5-HT1A autoreceptor also activates GIRK potassium channels to inhibit neuronal firing [86-89], and inhibits voltage-gated calcium channel activity to reduce calcium entry [90-92] (Figure 3). In post-synaptic cortical neurons, 5-HT1A-mediated inhibition of cAMP is thought to reduce CAMKII activity and to reduce AMPA receptor levels [93]. In hippocampal neurons, 5-HT1A receptor activation leads to activation of Akt and inactivation of GSK3β [94-96], which is also seen in raphe cultures [97], but has no effect on basal ERK1/2 phosphorylation [95,97]. Using rat raphe RN46A cells, a model of serotonergic neurons that express endogenous 5-HT1A autoreceptors [98,99], we found that 5-HT1A receptors signaled to inhibit of adenylyl cyclase and ERK1/2 phosphorylation, and that ERK1/2 inhibition was augmented upon differentiation of the cells to a serotonergic phenotype [100]. Similarly, in cortical neurons and human neuronal cultures, the 5-HT1A receptor also inhibits ERK1/2 activation [101,102]. Thus, modulation of ERK1/2 activity may be cell- and maturation-dependent and may also depend on the expression of negative regulators of this pathway (such as MKP or PP2A) [103,104]. Therefore, in the raphe, the signaling of the 5-HT1A autoreceptor appears to be largely inhibitory.

### 5-HT1A receptors and antidepressant response

Although antidepressants such as SSRIs, rapidly block 5-HT reuptake, chronic 3-wk treatment is required for clinical improvement and desensitization of the 5-HT1A autoreceptor has been implicated in this delay (Figure 3). Blier, de Montigny and colleagues demonstrated that multiple antidepressant treatments reduce auto-inhibition of serotonergic activity in the raphe nuclei by 5-HT1A autoreceptors, and in some cases sensitize responses of post-synaptic 5-HT1A receptors [105]. This work has led to the concept that chronic desensitization of the 5-HT1A autoreceptor is necessary to achieve increased serotonergic neurotransmission following antidepressant treatment [106-109] and to increase serotonin synthesis [110]. Consistent with this, the level of 5-HT1A autoreceptors is negatively correlated with raphe 5-HT synthesis in human subjects [74]. The prolonged time course of 5-HT1A desensitization is consistent with adaptive reduction in 5-HT1A receptor expression, as opposed to more rapid forms of desensitization such as receptor uncoupling or internalization. In studies of normal animals chronically treated with SSRIs, only acute desensitization of the autoreceptor is observed, with no changes in 5-HT1A autoreceptor RNA or protein levels in the raphe [111-113]. In contrast, studies in chronically stressed or older animals [114] have shown reductions in 5-HT1A RNA in raphe nuclei following chronic antidepressant treatment. During chronic SSRI treatment, 5-HT1A autoreceptors desensitize [107,115-119], leading to reduced 5-HT1A autoreceptor levels in animal depression models and depressed subjects [110,120-125], and restoration of raphe firing activity and 5-HT release. Thus both acute and chronic desensitization processes appear to be required for long-term inactivation of 5-HT1A autoreceptors in SSRI treatment of depressed animal models. However, the mechanisms involved in the preferential down-regulation of 5-HT1A autoreceptors, but not post-synaptic 5-HT1A receptors following chronic antidepressant treatment remain to be clarified.

Upon treatment with SSRIs, it is presumed that as well as inhibiting 5-HT reuptake at the nerve terminals in target brain regions, 5-HT levels are increased at the cell body and dendrites of raphe neurons leading to autoinhibition (Figure 2). However, the mechanism by which SSRI treatment enhances 5-HT release in the raphe is unclear. Although recurrent collaterals or intra-raphe cross-innervation occurs in culture [126], it has been difficult to demonstrate 5-HT terminals in the raphe nuclei [112]. The finding that VMAT2 is targeted to dendrites for somatodendritic vesicular dopamine release [127-129] provides a new mechanism by which 5-HT may be released somatodendritically. Recent evidence suggests that somatodendritic 5-HT release can occur, at least in culture [130]. But can transcriptional down-regulation account for a 3-week delay in antidepressant response? In cultured cells, siRNA can reduce target RNA within days, however, the half-life of 5-HT1A receptor protein *in vivo *is several days following doxycycline-induced suppression [131,132]. Furthermore, unlike in culture where signaling to down-regulation is immediate, 5-HT1A-mediated signaling to transcriptional down-regulation is gradual *in vivo *since 5-HT release is auto-inhibited and only gradually increases as autoreceptors become more desensitized, a process that may take weeks.

### 5-HT1A receptors in animal models of depression

Due to the limitations of heterogeneity in clinical samples and in the validity of animal models, our understanding of depression remains incomplete [30,133-135]. Nevertheless, mouse models have provided valuable insights into the role of 5-HT1A receptors in depression and anxiety. Importantly, 5-HT1A-null mice display increased anxiety behaviours [136-138] and are unresponsive to selective 5-HT reuptake inhibitor (SSRI) [139]. Conversely, global over-expression of the 5-HT1A receptor or enhancement of its post-synaptic signalling decreases anxiety [96,140]. Rescue of post-synaptic 5-HT1A receptor expression in early postnatal forebrain restores a normal anxiety phenotype, while its inhibition from postnatal day 13-34 induces anxiety in the adult, suggesting it has a role in development of the anxiety phenotype [131,141]. 5-HT1A-dependent inhibition of CAMKIIα appears to mediate the anxiety phenotype, since 5-HT1A-null mice with reduced CAMKII activation did not develop anxiety [141]. Recently, mice with a 30% decrease in 5-HT1A autoreceptors displayed increase in 5-HT neuron firing rate and augmented 5-HT release, and reduced depression-like behavior but no change in anxiety [132]. Hence, reduced activity of post-synaptic 5-HT1A receptors is implicated in anxiety, while an increased transcription of 5-HT1A autoreceptors associates with depression and resistance to chronic SSRI treatment [142].

## Transcriptional Regulation of the 5-HT1A Receptor Gene

**Figure 4:**
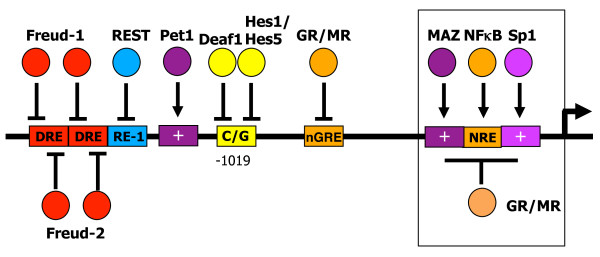
**Identified promoter elements of the human 5-HT1A receptor gene (HTR1A)**. The location of identified DNA elements on the 5-HT1A 5' regions flanking the start of translation (bold arrow) are shown figuratively. Identified activators (arrows) or repressors (bars) of transcription are also shown. Within the minimal promoter (box) there are GC-rich Sp1 and MAZ elements (+); NFkB response element (NRE); and glucocorticoid receptors (GR/MR) that inhibit transcription by blocking Sp1. Further upstream in a repressor/enhancer regions, a negative glucocorticoid response element (nGRE) also mediates direct GR/MR-induced repression. Hes and Deaf1 proteins repress the 5-HT1A promoter at the C(-1019) allele, while in serotonin neurons Pet-1 exerts strong enhancer activity. A strong repressor region that silences expression in 5-HT1A-negative non-neuronal cells, but also represses in 5-HT1A-positive neuronal cells is located upstream that includes elements for REST (RE-1), Freud-1 and Freud-2 (DRE).

Because acute desensitization (uncoupling and internalization) occurs rapidly (sec-min) and is rapidly reversible [143,144], we postulated that reduced transcription of 5-HT1A autoreceptors could account for the three-week delay in clinical response following antidepressant treatment [108,142,145]. Supporting this hypothesis, transgenic mice with only 30% repression of 5-HT1A autoreceptors display an enhanced and rapid response to SSRIs [132], suggesting that transcriptional repression of the 5-HT1A autoreceptor could be key to an effective antidepressant response. In characterizing the 5-HT1A promoter, we have uncovered a number of important regulators at the minimal promoter, upstream repressor and enhancer regions, as well as at a C(-1019)G polymorphism, that could affect 5-HT1A autoreceptor expression (Figure 4).

### 5-HT1A minimal promoter

To examine the transcriptional regulation of the 5-HT1A receptor gene, the promoter region has been characterized using transcriptional reporter assays in cultured cell lines [99,108,146]. Most non-neuronal cell lines and tissues express low or undetectable levels of 5-HT1A receptors [61], while some neuronal cell lines express endogenous 5-HT1A receptors. In particular, the rat raphe RN46A cells are serotonergic and express 5-HT1A receptors, and can serve as a model for 5-HT1A autoreceptor regulation [99]. While several non-serotonergic neuroblastoma cells (human SKN-SH, rodent NG108-15 and SN-48 cells) also express 5-HT1A receptors and model post-synaptic 5-HT1A receptors [147-149]. The human and mouse 5-HT1A promoters lack a TATA box and have multiple transcription start sites, while the rat 5-HT1A promoter has a major TATA-driven start site [99,146]. Nevertheless, the minimal promoter located within 300 bp upstream of the intronless coding sequence is highly conserved. This minimal promoter is typical of a "housekeeping" promoter and consists of a series of GC-rich enhancer elements recognized by the ubiquitous factors MAZ1 and Sp1 [146] that drive non-selective expression of the gene in all cell types, whether they express endogenous 5-HT1A receptors or not [99]. Within the minimal promoter, there is a conserved NFkB response element that may mediate induction of 5-HT1A expression by NFkB [150]. NFkB is observed in several types of immune cells including B- and T-lymphocytes, neutrophils and macrophages in which basal 5-HT1A receptor levels are very low, but can be greatly induced by mitogenic stimulation to augment the mitogenic response [151-153]. Finally, there is evidence that suppression of 5-HT1A receptor expression by glucocorticoids is mediated by inhibitory actions on the minimal promoter via Sp1 and NFkB elements [154].

### 5-HT1A repressor region

Located upstream from the minimal promoter is a series of repressor elements that silence 5-HT1A expression in non-neuronal cells, but also repress 5-HT1A transcription in neuronal cell lines that express 5-HT1A receptors [155,156]. Importantly these elements are conserved between human, rat and mouse genes and are functional. One of the key elements is a consensus repressor element-1 (RE-1) site that is recognized by REST/NRSF, a key pan-neuronal repressor of multiple neuronal genes [157-159]. REST has been shown to be crucial for silencing neuronal gene expression in neural stem cells or progenitors and non-neuronal cells, but is down-regulated upon neuronal differentiation, allowing for expression of neuronal genes. However, the 5-HT1A receptor is not expressed in all neuronal subtypes, hence additional repressors are required to restrict its expression to appropriate neurons. Located adjacent to the RE-1 site, we identified a dual repressor element (DRE, 31-bp) that mediates the strongest repression of the 5-HT1A promoter (Figure 4) [156]. In non-neuronal cells two nuclear protein complexes bind the DRE, one at the 5' end (FRE), and the other at the 3' end (TRE); however in raphe RN46A cells only the FRE-complex is present. In raphe RN46A cells, mutation of the FRE de-repressed 5-HT1A transcription by 10-fold, whereas in non-neuronal cells, deletion of the entire DRE was required. Using a yeast one-hybrid cloning approach Freud-1/CC2D1A (**FRE U**nder **D**ual repression binding protein) was identified as a protein that binds and represses at the FRE site [160]; subsequently, the homologue Freud-2/CC2D1B was identified as the second DRE-binding protein [161,162]. Freud-1 and Freud-2 are colocalized with 5-HT1A receptors in neurons, where they play complementary roles to regulate the level of 5-HT1A receptor expression. In particular, Freud-1, but not Freud-2, is strongly expressed and colocalized with 5-HT and 5-HT1A receptors in raphe nuclei, while both Freud-1 and Freud-2 are colocalized with the receptor in post-synaptic areas such as cortex and hippocampus [161,163]. Together, Freud-1 and Freud-2 mediate dual repression of 5-HT1A receptor expression in most cell types including many post-synaptic neurons to restrict 5-HT1A receptor expression to appropriate neurons. Based on its role in RN46A cells and its localization in 5-HT neurons *in vivo*, Freud-1 appears to be the dominant repressor of 5-HT1A autoreceptor expression. However, it is possible that in mental illness, other repressors may play a role. For example, REST expression is up-regulated in serotonergic raphe cells from depressed suicide as compared to control brains, and may restrain over-expression of 5-HT1A autoreceptors observed in these subjects [164].

Located between the minimal promoter and the upstream DRE is a region that exhibits both enhancer and repressor activities (Figure 4). Within this region, a novel type of negative glucocorticoid response element (nGRE), composed of two GRE half-sites separated by 6 nucleotides (rather than 3 nucleotides as for a typical consensus GRE) [165] was identified. The nGRE is conserved between human, mouse and rat, although its function has only been demonstrated for the rat nGRE thus far. The 5-HT1A nGRE mediates synergistic repression by both high and low affinity glucocorticoid receptors (MR and GR), suggesting a key role in repression of 5-HT1A receptors in the hippocampus, in which both these receptors are present [166]. In the raphe, only GR has been detected, and it appears to have a relatively weaker ability to suppress 5-HT1A receptor expression compared to hippocampus [167-171]. Interestingly, 5-HT1A agonists downregulate GR in raphe cells in culture, which would lead to an increase in 5-HT1A autoreceptors [172]. Conversely, knockdown of GR abrogates the down-regulation of 5-HT1A autoreceptors induced by chronic mild stress in mice [173]. Since glucocorticoids are dys-regulated in depression, the chronic elevation of cortisol may ultimately desensitize GR and could contribute to increase 5-HT1A autoreceptor expression in depression.

### C(-1019)G HTR1A polymorphism (rs6295)

**Figure 5:**
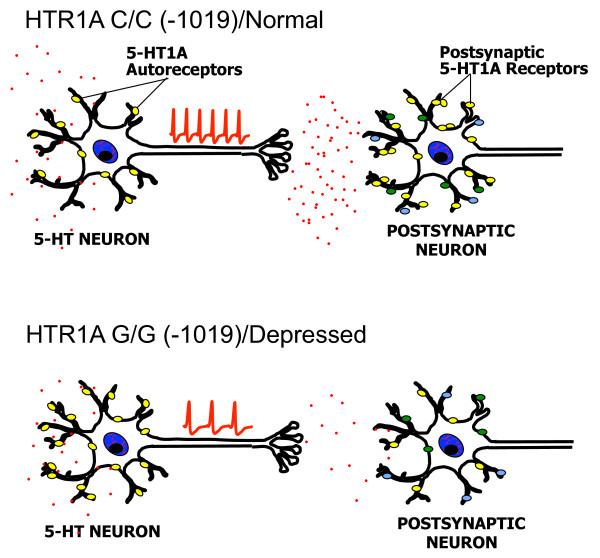
**Proposed actions of 5-HT1A C(-1019)G genotype in normal vs. depressed subjects**. In normal subjects containing homozygous HTR1A C/C (-1019) genotype, 5-HT neurons fire at their normal rate (red action potentials), releasing 5-HT (red dots) at the synapse for activation of receptors (ovals), and to a lesser degree at the raphe cell body to restrain neuronal activity by negative feedback through the 5-HT1A autoreceptor (yellow ovals). In the G/G genotype and in depressed subjects, 5-HT1A autoreceptor expression is increased which would reduce neuronal firing and 5-HT release, while post-synaptic 5-HT1A receptors are reduced in certain regions, which would decrease response to 5-HT release. Normal subjects with the G/G genotype have a lesser increase of 5-HT1A autoreceptors, and are able to compensate for the effect of the G/G genotype on transcription.

In our analysis of the 5-HT1A promoter we identified a human C(-1019)G 5-HT1A polymorphism located within the repressor/enhancer region. The G allele and G/G genotype were associated with major depression and suicide [174,175]. Since the C(-1019)G polymorphism is located in a 26-bp palindrome, we addressed whether this palindrome could bind protein in nuclear extracts of raphe RN46A cells, and showed a specific complex that preferentially recognized the C-allele over the G-allele. Using a yeast one-hybrid screen, Deaf1 (NUDR) and Hes5 were identified as repressors of the C- but not the G-allele of the 5-HT1A promoter (Figure 4). Deaf1 (Deformed autoregulatory factor-1) binds to a minimal TCG consensus sequence [176] present in the human, rat and mouse 5-HT1A genes [175] and can act as a repressor or enhancer [176,177]. By supershift analysis, Deaf1 was detected as a major component of the RN46A nuclear protein C-allele palindrome-binding complex. When transfected in RN46A cells, Deaf1 suppressed 5-HT1A receptor transcription, and reduced 5-HT1A RNA and protein expression. However, unlike Freud-1, while mutation of the palindrome blocked Deaf1 repression, it did not de-repress basal 5-HT1A transcription compared to mutation of the FRE, suggesting that Freud-1 is the predominant repressor of the autoreceptor in RN46A cells. In the adult rat and human brain, Deaf1 is colocalized with 5-HT1A receptors, and in the raphe nuclei it is also colocalized with 5-HT [175,178]. In other neuronal 5-HT1A-expressing cell types, instead of repressing 5-HT1A gene expression as seen in raphe or non-neuronal cells, Deaf1 enhances 5-HT1A expression, and the G-allele reduces basal 5-HT1A expression [148]. Based on this dual activity of Deaf1, the G/G(-1019) genotype is expected to increase 5-HT1A autoreceptor levels to reduce 5-HT neuron firing, and decrease post-synaptic 5-HT1A receptors, thereby synergistically reducing 5-HT neurotransmission (Figure 5). In support of this, depressed subjects homozygous for the 5-HT1A G/G(-1019) genotype have increased 5-HT1A autoreceptor binding potential [179-181], which is consistent with the increase in 5-HT1A autoreceptors observed in post-mortem studies of depressed suicides [46-48]. Thus, the 5-HT1A G(-1019) allele may alter 5-HT1A receptor expression *in vivo *by blocking Deaf1 function.

The Hes proteins, which also preferentially recognize the HTR1A C(-1019) allele, appear to play an important role in regulating early induction of the 5-HT1A autoreceptor. Hes5, and especially Hes1, repress neuronal gene expression in neural precursor cells and are down-regulated upon neuronal differentiation [182]. We therefore addressed whether Hes1 can repress 5-HT1A receptor expression, and found that Hes1 exerts even stronger repression than either Hes5 or Deaf1. However, Deaf1 appears to be the dominant factor when the two are co-expressed [183]. To address whether Hes1 influences 5-HT1A receptor expression *in vivo*, we examined embryonic midbrain tissue from the Hes1 -/- mice for 5-HT1A receptor RNA levels at the initiation of serotonergic neuronal differentiation. We found that 5-HT1A RNA was prematurely upregulated with an expanded distribution of midbrain expression. Thus, reduction in repression by both Deaf1 and Hes proteins at the G-allele of the 5-HT1A promoter could lead to upregulation of 5-HT1A autoreceptor expression beginning in early serotonergic differentiation and extending to adulthood.

The expression of Deaf1, Freud-1 and Freud-2 also appears to be dys-regulated in human depression in a region-specific manner [161,164,178,184]. In serotonergic raphe cells, both Deaf1 and REST are upregulated, and 5-HT1D receptor RNA is increased, and there is a trend for increased 5-HT1A and 5-HT1B RNA [164]. Hence, despite compensatory upregulation of repressors, there appears to be a general upregulation of 5-HT1 autoreceptor expression on serotonergic neurons. However, the G-allele would be expected to attenuate Deaf1 action on the 5-HT1A receptor gene.

### Pet-1 enhancer elements

In addition to repression, 5-HT1A autoreceptor expression is subject to regulation by enhancers, the most important of which appears to be Pet-1. Pet-1 was identified as a critical regulator of 5-HT marker genes including TPH, 5-HTT, and ADC genes [185], and knockout of Pet-1 results in a substantial loss of serotonin in the brain, although a few 5-HT neurons appear to persist [186]. Hence we addressed whether Pet-1 regulates 5-HT1A receptor gene transcription [187]. There are several putative Pet-1 sites located in the 5-HT1A promoter, and deletion analysis demonstrated that while all of the sites have some activity, deletion of the upstream Pet-1 site located at -1406 bp reduced 5-HT1A promoter activity by over 90%, indicating a predominant role for this site. Consistent with a role for Pet-1 in regulation of 5-HT1A autoreceptor expression *in vivo*, the Pet-1 knockout mice demonstrated a nearly complete loss of 5-HT1A RNA and protein specifically in the raphe [187-189], while at post-synaptic target tissues 5-HT1A expression was modestly affected [187]. Thus, Pet-1 functions as a major enhancer of 5-HT1A autoreceptor expression. However, since Pet-1 is also required for TPH2 expression, blocking Pet-1 would actually reduce 5-HT neurotransmission as shown in the Pet-1 knockout mice, which leads to an anxious and aggressive behavioral phenotype [186].

In summary, we have identified several key transcription factors, including REST, Freud-1, GR, Deaf1, Hes1 and Pet-1, which coordinately regulate 5-HT1A autoreceptor expression and its modulation by glucocorticoids, 5-HT, and other neurotransmitters.

## 5-HT1A Autoreceptors in Human Depression

Given the role of 5-HT1A autoreceptors in regulation of the serotonin system and the importance of 5-HT in clinical depression, several approaches have addressed whether 5-HT1A receptor expression is altered in depression. In depressed subjects, the observed increases in 5-HT1A autoreceptors could be due to increased 5-HT1A autoreceptor transcription, while region-specific reductions in post-synaptic 5-HT1A receptors could result from reduced transcription in these regions. These changes in 5-HT1A receptors would result in a global reduction in 5-HT neurotransmission, and predisposition to depression (Figure 5). In agreement with this idea, the G(-1019) 5-HT1A allele, which leads to increased 5-HT1A autoreceptor transcription, has been associated with major depression and suicide [175], and this association has been replicated and extended in most [174,179,181,190-197], but not all studies [198]. The 5-HT1A G(-1019) allele has also been associated with anxiety [199-202]. Importantly, the 5-HT1A G/G genotype is associated with increased 5-HT1A autoreceptors in depressed subjects [179-181], suggesting that 5-HT1A G(-1019) allele is a risk factor for depression by increasing 5-HT1A autoreceptor levels to reduce 5-HT neurotransmission [108,145]. Interestingly, studies in normal subjects have not found an association of the G/G genotype with depressed or anxious mood [203]. Furthermore, in normal subjects, although a trend for increased 5-HT1A autoreceptor levels with the G/G genotype is observed, it was not statistically significant [204]. These results suggest that although the G-allele may promote higher expression of 5-HT1A autoreceptors (Figure 5), normal subjects are able to compensate for the effect of the G allele, while depressed patients are not. For example, the absolute level of 5-HT1A receptors may differ between depressed and normal subjects due to differences in the regulation of 5-HT1A expression (e.g., by increased glucocorticoids) or in the expression of 5-HTT, TPH2, MAO or other genes that influence the amount of 5-HT that is present extracellularly in the raphe [205,206] and would indirectly affect the level of 5-HT1A autoreceptors through regulating autoreceptor desensitization. In addition, it is possible that impairment of Deaf1 action could account for increased levels of 5-HT1A autoreceptors in G/G subjects, and could also mediate a reduction in post-synaptic 5-HT1A receptors, suggesting Deaf1 as a potentially important mediator of transcriptional dys-regulation of the 5-HT1A receptor gene in depression.

An increase in 5-HT1A autoreceptor levels delays or prevents antidepressant response [132] and 3 weeks of treatment with antidepressants is required for clinical improvement, due to recurrent inhibition of raphe activity by the 5-HT1A autoreceptor (Figure 2). One strategy to overcome negative feedback by 5-HT1A autoreceptor has been to use 5-HT1A partial agonists such as pindolol or buspirone, to block or desensitize the autoreceptor and accelerate SSRI action [207-209]. However, these compounds have insufficient specificity since they affect both pre-and post-synaptic receptors, and display only partial specificity for 5-HT1A autoreceptors [210,211]. We and others have found that the G(-1019) allele associates with reduced antidepressant response [174,212-214], suggesting that regulation by Deaf1 could facilitate antidepressant response. Successful treatment of panic disorder patients with chronic SSRIs correlates with a normalization of pre- and post-synaptic 5-HT1A receptors, suggesting that down-regulation of pre-synaptic receptors concomitant with an up-regulation of post-synaptic 5-HT1A receptors may be critical for treatment response [45,215]. Among the transcriptional regulators of the 5-HT1A promoter, Deaf1 is of particular interest since it displays repressor activity on 5-HT1A autoreceptor expression, but enhancer activity on post-synaptic 5-HT1A receptors, a combination of activities that would normalize pre- and post-synaptic 5-HT1A receptor levels to enhance serotonergic neurotransmission.

## Conclusion

In conclusion, since dys-regulation of the 5-HT1A autoreceptor has the potential to affect the activity of the entire 5-HT system, it is critical to identify the transcriptional mechanisms underlying its long-term regulation. Understanding the transcription factors involved may provide important clues to the molecular mechanisms responsible for 5-HT1A receptor dys-regulation in mental illness. Transcriptional regulators of the 5-HT1A autoreceptor may also constitute important targets to restore normal levels of the receptor and improve treatment outcome for depression and related mental illnesses.

## Competing interests

The authors declare that they have no competing interests.

## Authors' contributions

PA conceived of the topic, searched the literature and drafted the manuscript. BF and AM provided specific sections and corrections. All authors read and approved the final manuscript.
